# Enhancing ablation outcomes: vein of Marshall ethanol infusion in persistent atrial fibrillation with severe left atrial enlargement

**DOI:** 10.3389/fcvm.2025.1554321

**Published:** 2025-04-10

**Authors:** Tao Luo, Tao Liu, Bo Cui, Chenggang Deng, Yanhong Chen, Xiong Xiong, Jinlin Zhang, Gang Wu

**Affiliations:** ^1^ Department of Cardiology, Renmin Hospital of Wuhan University, Wuhan, Hubei, China; ^2^ Cardiovascular Research Institute, Wuhan University, Wuhan, Hubei, China; ^3^ Hubei Key Laboratory of Cardiology, Wuhan, Hubei, China; ^4^ Department of Cardiology, Wuhan Asia Heart Hospital, Wuhan, Hubei, China

**Keywords:** vein of Marshall, ethanol infusion, radiofrequency ablation, persistent atrial fibrillation, left atrial enlargement

## Abstract

**Background:**

Left atrial (LA) enlargement is a widely recognized factor that increases the risk of ablation failure in patients with atrial fibrillation (AF). This retrospective, observational study aimed to assess the influence of vein of Marshall (VOM) ethanol infusion (VOM-EI) on persistent atrial fibrillation (PsAF) ablation among patients with severe LA enlargement.

**Methods:**

In this research, 178 patients underwent the VOM-EI followed by the radiofrequency (RF) ablation procedure, which was based on circumferential pulmonary vein antrum (CPVA) ablation and linear ablation of the roof of the LA and the mitral isthmus (MI). In comparison, 83 patients only underwent the RF ablation procedure.

**Results:**

In the VOM + RF ablation group, the duration of left CPVA ablation was shorter compared to the RF ablation group (20.3 ± 8.7 minutes vs. 27.1 ± 8.1 minutes, *P* < 0.0001). The rate of MI block was higher (94.9% vs. 85.5%, *P* = 0.01) in the VOM + RF ablation group, with a shorter MI ablation time (23.2 ± 10.8 minutes vs. 30.5 ± 11.8 minutes, *P* < 0.0001), and a lower need for coronary sinus vein ablation compared to the RF ablation group (64.0% vs. 78.3%, *P* = 0.02). Throughout the one-year observation period, the VOM + RF ablation group exhibited a notably superior survival rate without recurrence compared to the RF ablation group (78.7% vs. 65.1%, *P* = 0.02). As compared to the RF ablation group, the VOM + RF ablation group had a lower rate of recurrence with atrial tachycardia (8.4% vs. 19.3%, *P* = 0.01).

**Conclusion:**

The VOM-EI facilitated the left CPVA and the MI ablation and improved the ablation outcomes in patients with severe LA enlargement for treating PsAF.

## 1 Introduction

It is safe and more effective than antiarrhythmic drugs (AADs) to treat atrial fibrillation (AF) with catheter ablation (1). Although pulmonary vein isolation (PVI) is extensively employed for AF ablation, its efficacy is consistently inadequate when conducted as a standalone procedure in patients with persistent atrial fibrillation (PsAF) (2–4). Left atrial (LA) linear ablation beyond PVI is recommended in patients with PsAF, although the benefits remain disputable (5–7). The research findings indicate that the combined linear ablation strategy, including mitral isthmus (MI) linear ablation, is ineffective in improving the short-term and long-term outcomes of patients with long-standing PsAF. The CASA-AF randomized controlled trial demonstrated that the standard catheter ablation strategy, including PVI, roof line, and inferior line to create a posterior wall box lesion, and the MI line, showed no difference compared with thoracoscopic ablation. Both strategies yielded poor outcomes, with rates of no AF or AT occurrence at 12 months after surgery of 28% and 26%, respectively (8). Long-term follow-up showed that in the standard catheter ablation strategy group with a single surgery and without the use of anti-arrhythmic drugs, 7 patients (12%) had no recurrence of AF or AT at 36 months, while in the thoracoscopic ablation group, 5 patients (11%) had no recurrence of AF or AT at 36 months (9). The vein of Marshall (VOM) is an authentic atrial vein that connects with the underlying myocardium, consisting of myocardial connections, arrhythmogenic foci, and innervation. It is located next to the MI, which can be treated with ethanol (10). In a randomized clinical trial (11), it was demonstrated that the VOM ethanol infusion (VOM-EI) enhanced the effectiveness of PsAF ablation, as evidenced by an improved success rate. LA enlargement is a widely recognized factor that increases the risk of ablation failure in patients with AF (4, 12, 13). A multicenter retrospective observational study was conducted to evaluate the influence of VOM-EI on treating PsAF in patients with severe LA enlargement.

## 2 Materials and methods

### 2.1 Study participants

The retrospective observational study included patients who were sequentially enrolled from two AF centers, namely Wuhan Asia Heart Hospital and Renmin Hospital of Wuhan University, during the period from January 2021 to August 2022. Study approval was obtained from Wuhan Asia Heart Hospital's Ethics Committee. The inclusion criteria for severe LA enlargement were LA anterior-posterior diameter over 45 mm measured by transthoracic echocardiogram. The study included both PsAF and long-standing PsAF that met clinical guidelines (1). PsAF Definition: Episodes of AF that last for more than seven days, as well as those terminated by cardioversion after seven days of symptoms. Long-standing PsAF Definition: Episodes of AF that last for more than one year when a rhythm control strategy is decided upon. A prior history of AF ablation or valvular AF was excluded from the study.

### 2.2 Study design

The enrolled population was grouped according to the ablation strategies received. The radiofrequency (RF) ablation group: Bilateral circumferential pulmonary vein antrum (CPVA) ablation was performed initially to achieve PVI. Linear ablation of the LA roof and the MI were performed to achieve a bidirectional block after PVI. If atrial tachycardia (AT) occurred, ablation guided by activation mapping was performed. The cavotricuspid isthmus (CTI) was linearly ablated in patients showing typical atrial flutter. The choice to carry out complex fractionated atrial electrogram (CFAE) ablation and/or LA posterior wall isolation (PWI) was left to the operator's discretion. Sinus rhythm (SR) was restored through direct-current cardioversion after the aforementioned ablation procedure failed to resolve AF. The VOM + RF ablation group: The VOM-EI preceded the RF ablation procedure.

### 2.3 Periprocedural management

Electrocardiogram (ECG), Holter ECG monitoring, and transthoracic echocardiogram were available. Amiodarone should be discontinued at least two weeks before AF ablation, and other Antiarrhythmic drugs (AADs) should be discontinued for at least five half-lives. A transesophageal echocardiogram was performed immediately before the ablation procedure to rule out thrombosis in the left atrium. The administration of a non-vitamin K antagonist oral anticoagulant (NOAC) or a vitamin K antagonist (VKA) was discontinued on the day of the procedure and then recommenced after a delay of 4–6 hours while ensuring that pericardial effusion was excluded. Intravenous heparin was administrated to maintain an active coagulation time of 300–350 s during the procedure.

### 2.4 General anesthesia

All patients underwent general anesthesia. After endotracheal intubation, intravenous etomidate, remifentanil, and benzene sulfonyl-atracurium were used to induce anesthesia. After that, intravenous dexmedetomidine, remifentanil, and sevoflurane were inhaled. A bispectral index was used to measure sedation level and consciousness level.

### 2.5 VOM-EI procedure

**Figure 1:**
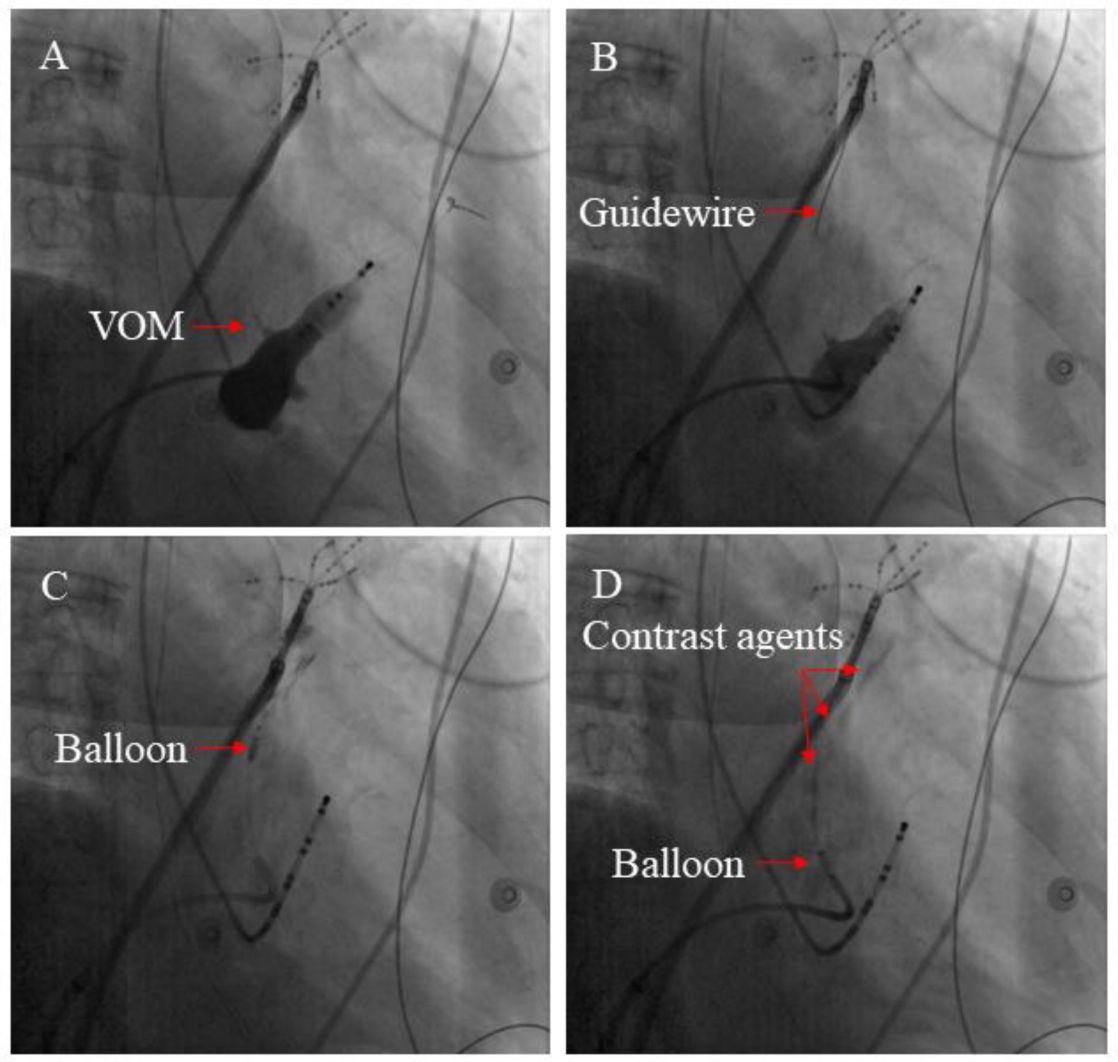
The main steps involved in the VOM-EI process. **(A)** An angiography of the CS vein demonstrated the presence of the VOM (see red arrow). **(B)** By inserting a guidewire (shown by the red arrow), the distal section of the VOM could be accessed. **(C)** Anhydrous ethanol was injected through the balloon catheter inflated at the distal segment of the VOM (red arrow indicating inflated balloon catheter). **(D)** The ablated myocardium can be colored with contrast agents by multiple injections of anhydrous ethanol (The red arrow indicates the contrast agents).

A previous study described the VOM-EI procedure in detail (14). A pre-molded Judkins Right 3.5 guide catheter (Cordis) was introduced into the CS under the guidance of a Runthrough NS guide wire (Terumo), and angiography was performed to visualize VOM. The Judkins Right 3.5 guide catheter was adjusted to make its tip enter or close to the proximal end of VOM, and the Runthrough NS guide wire was adjusted to make its tip located at the distal end of VOM. Under the guidance of Runthrough NS wire, the OTW balloon catheter (Boston Scientific) was placed into the distal VOM, dilated, and an iodine contrast agent was injected into it to confirm the blockage of the distal VOM blood flow. Slowly inject about 3–6 ml of absolute alcohol through the OTW balloon catheter, then withdraw the OTW balloon catheter to the proximal segment of VOM, and slowly inject about 3–6 ml of absolute alcohol according to the previous steps. Myocardial staining around VOM can be observed by performing angiography (Figure 1).

### 2.6 LA voltage mapping

**Figure 2:**
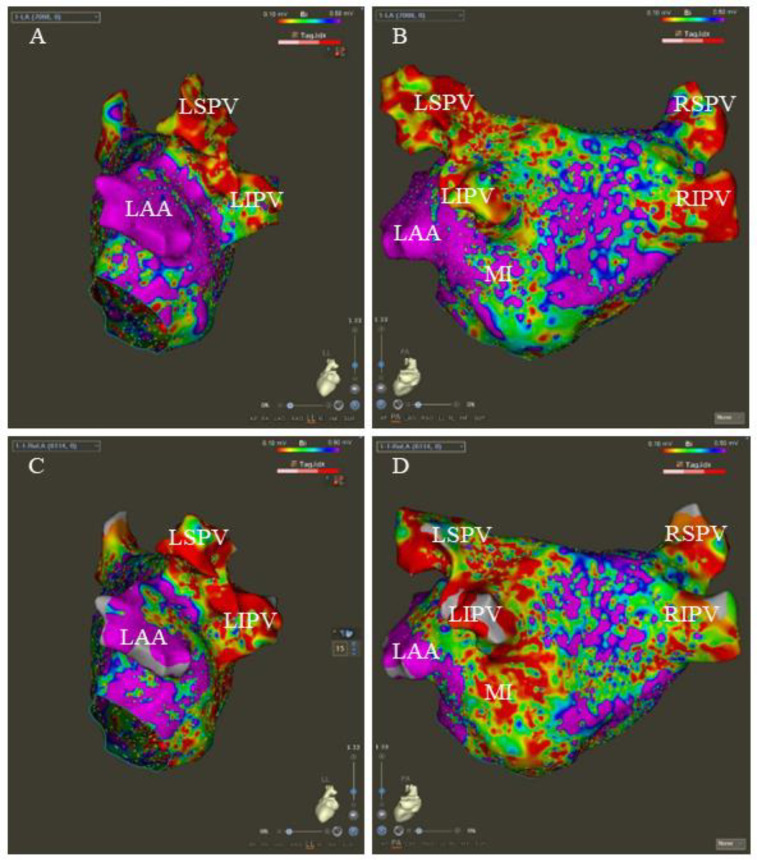
The typical LA voltage maps at baseline and after the VOM-EI procedure. The baseline LA voltage in left lateral (LL) and posteroanterior (PA) views, respectively **(A,B)**. After the VOM-EI procedure, the LA voltage shows a larger LVA, mainly located in the left atrial appendage (LAA)- the left inferior pulmonary vein (LIPV), the posterior wall of the left pulmonary vein, and the mitral isthmus (MI) in LL and PA views, respectively **(C,D)**. LSPV stands for left superior pulmonary vein, RSPV stands for right superior pulmonary vein, and RIPV stands for right inferior pulmonary vein.

LA's three-dimensional electrical reconstruction and voltage map were obtained using a high-density mapping catheter (Pentaray, Biosense Webster). All patients underwent baseline LA voltage mapping, and patients from the VOM + RF ablation group underwent voltage mapping on the ethanol-ablated area following the VOM-EI procedure. The voltage color scale was set from 0.1 to 0.5 mV. Bipolar peak-to-peak voltage below 0.5 mV was considered a low voltage area (LVA) (15). Figure 2 illustrates the typical LA voltage maps at baseline and after the VOM-EI procedure.

### 2.7 RF ablation procedure

**Figure 3:**
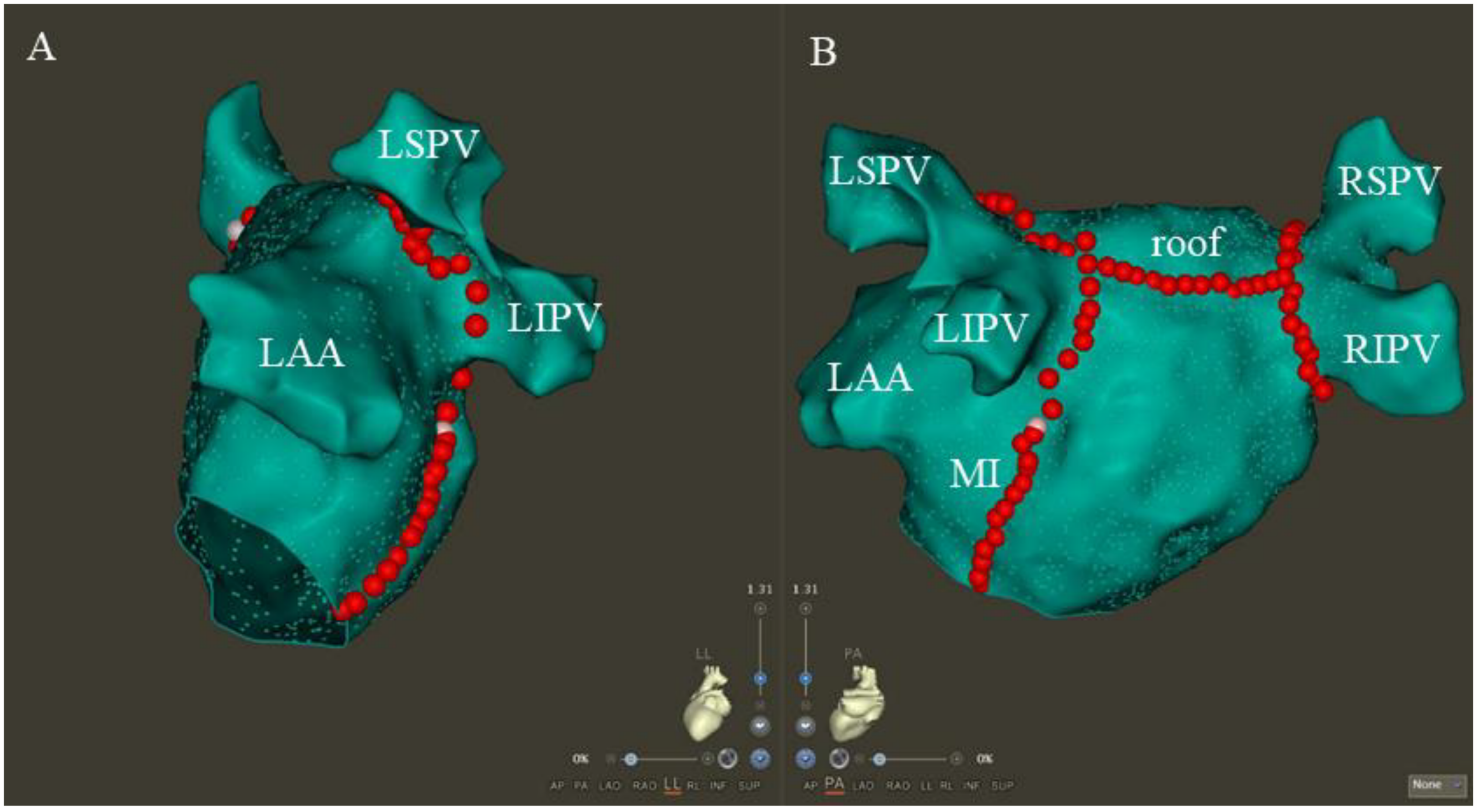
The typical LA maps after CPVA ablation, roof ablation, and posterior MI ablation. Following CPVA and linear ablation of the roof and posterior MI, the typical LA maps are shown in the left lateral and posteroanterior views, respectively **(A,B)**. The abbreviations LSPV, RSPV, LIPV, and RIPV correspond to the left superior, right superior, left inferior, and right inferior pulmonary veins, respectively, with MI and LAA standing for the mitral isthmus and left atrial appendage.

RF ablation was conducted using a saline infusion catheter (ST SF, Biosense Webster), with the power control mode set at 45W, 15 ml/min, and 43°C. CPVA and linear ablation were carried out in accordance with the ablation index (AI) (16). The AI priority was 350 for the posterior wall, 450 for the anterior wall, 500 for the MI line, 400 for the roof line, and 450 for the CTI line. We ablated the CPVA in accordance with the CLOSE protocol, and we validated the presence of PVI at the entry and exit blocks (17). A continuous ablation was performed on the roof between the two superior pulmonary veins in a linear fashion (18). In pacing the left atrial appendage, a shorter activation time was observed below the roof line, confirming the roof line block. The posterior MI line was successfully established by successive ablation from the mitral valve edge to the inferior pulmonary vein ostium. In order to confirm the bidirectional block, a validated differential pacing technique was used (19). CS vein ablation was performed (25 W, 15 ml/min, 43℃) if the endocardial ablation did not achieve MI block (20). CTI lines were formed by ablating the lower right atrium from the inferior vena cava to the tricuspid valve. With a simplified differential pacing method, the CTI block could be confirmed (21). CFAE ablation was performed by ablating all consecutive electrograms with an average circumference of less than 120 ms (22). After achieving PVI and linear block of the roof, LA PWI was achieved by creating a continuous ablative lesion between the two inferior pulmonary veins by point-by-point ablation. Achieving PWI was confirmed by the presence of an exit block during posterior wall pacing (23). Figure 3 shows the customary LA maps after CPVA ablation, roof ablation, and posterior MI ablation.

### 2.8 Postprocedural management

Proton pump inhibitors were administered for a month to prevent esophageal mucous membrane impairment. Anticoagulants were administered for at least three months based on the CHA2DS2VASc score.

### 2.9 Follow up

The Holter ECG recording should be completed three months and one year after the procedure, as well as whenever suspicions of arrhythmia-related symptoms arise. An episode of AF or atrial tachycardia (AT) lasting more than 30 s during follow-up was considered a recurrence. Following the procedure, AAD was prioritized for treating recurrent arrhythmias during a blank period of three months. Recurrence after the blank period, or re-ablation during the blank period due to AAD therapy failure, was considered a recurrence of AF.

### 2.10 Statistical analysis

Categorical variables were described by percentage and analyzed using the Fisher exact test. Student's *t*-test was used to analyze continuous standard distribution variables represented by mean ± standard deviation (SD). Kaplan–Meier curves were plotted to reflect survival without recurrence, and the log-rank *p*-test was used to analyze the differences between the two groups. Statistics were considered significant when *p* < 0.05 (double-tailed).

## 3 Results

### 3.1 Baseline characteristics

In this research, of the 197 patients who met the enrollment criteria and completed follow-up, 19 (9.6%) patients were excluded from the study because the VOM-EI procedure could not be completed, including 15 patients whose VOM could not be identified by angiography, 4 patients could not successfully undergo the procedure because the VOM vessels were too small or too twisted, and 178 (90.4%) patients were eventually enrolled in the VOM + RF ablation group. In total, 261 patients were included in the study: 178 underwent VOM + RF ablation, and 83 underwent RF ablation. The demographics, comorbidities, and clinical data at baseline did not differ statistically significantly between the two groups (Table 1).

**Table 1:** Baseline characteristics.

Characteristic	VOM + RF ablation (*n* = 178)	RF ablation (*n* = 83)	*p*-value
Age, years	61.3 ± 7.5	61.5 ± 8.7	0.85
Male sex, *n* (%)	126 (70.8)	64 (77.1)	0.30
Body mass index	26.8 ± 2.8	26.9 ± 2.7	0.79
Heart failure with reduced ejection fraction, *n* (%)	30 (18.1)	12 (14.5%)	0.72
Hypertension, *n* (%)	107 (60.1%)	44 (53.0%)	0.29
Diabetes mellitus, *n* (%)	27 (15.2%)	15 (18.1%)	0.86
History of stroke, *n* (%)	20 (11.2%)	6 (7.2%)	0.38
PsAF, *n* (%)	74 (41.6%)	37 (44.6%)	0.69
Long-standing PsAF, *n* (%)	104 (58.4%)	46 (55.4%)	0.69
LA diameter, mm	48.9 ± 2.3	48.5 ± 2.5	0.20

Data presentation includes mean ± SD or absolute numbers along with percentages (n%). VOM, vein of Marshall; RF, radiofrequency; PsAF, persistent atrial fibrillation; LA, left atrium.

### 3.2 Procedural data

The two groups showed no significant difference in the baseline size of the left atrial LVA (5.3 ± 4.2 vs. 5.6 ± 4.7 cm^2^, *P* = 0.61). There was a significant increase in the total size of left atrial LVA after the VOM-EI procedure in the VOM + RF ablation group (18.8 ± 8.9 vs. 5.3 ± 4.2 cm^2^, *P* < 0.0001). Table 2 contains extensive details regarding ablation procedures. Compared to those in the RF ablation group, patients in the VOM + RF ablation group required a longer total procedural and fluoroscopy time (164.0 ± 20.5 minutes vs. 156.2 ± 20.3 minutes, *P* = 0.004, and 25.9 ± 6.2 minutes vs. 21.8 ± 6.1 minutes, *P* < 0.0001, respectively). The total procedural and fluoroscopy time required to complete VOM-EI were 27.7 ± 13.0 minutes and 6.7 ± 3.7 minutes, respectively. Although all patients achieved PVI, in the VOM + RF ablation group, the left CPVA ablation time was shorter than in the RF ablation group (20.3 ± 8.7 minutes vs. 27.1 ± 8.1 minutes, *P* < 0.0001). There was no significant difference in the LA roof block rate and ablation time between the two groups, which were 97.2% vs. 96.4%, *P* = 0.71, and 8.0 ± 2.1 minutes vs. 7.9 ± 2.2 minutes, *P* = 0.72, respectively. Compared with the RF ablation group, in the VOM + RF ablation group, the MI block rate was higher (94.9% vs. 85.5%, *P* = 0.01), the MI ablation time was shorter (23.2 ± 10.8 minutes vs. 30.5 ± 11.8 minutes, *P* < 0.0001), and required less CS vein ablation (64.0% vs. 78.3%, *P* = 0.02). There was no statistical difference between the two groups regarding the conversion rate to sinus rhythm or AT, the CTI ablation rate, the CFAE ablation rate, the PWI rate, or the cardioversion rate during the ablation procedure.

**Table 2:** Ablation procedural data.

Procedural data	VOM + RF ablation (*n* = 178)	RF ablation (*n* = 83)	*p*-value
Total procedural time, min	164.0 ± 20.5	156.2 ± 20.3	0.004
Total fluoroscopy time, min	25.9 ± 6.2	21.8 ± 6.1	<0.0001
Procedural time of VOM-EI, min	27.7 ± 13.0	–	
Fluoroscopy time of VOM-EI, min	6.7 ± 3.7	–	
Successful PVI, *n* (%)	178 (100)	83 (100)	>0.9999
Left CPVA ablation time, min	20.3 ± 8.7	27.1 ± 8.1	<0.0001
Right CPVA ablation time, min	27.8 ± 6.7	27.5 ± 7.2	0.46
LA roof block, *n* (%)	173 (97.2)	80 (96.4)	0.71
LA roof ablation time, min	8.0 ± 2.1	7.9 ± 2.2	0.72
MI block, *n* (%)	169 (94.9)	71 (85.5)	0.01
MI ablation time, min	23.2 ± 10.8	30.5 ± 11.8	<0.0001
CS vein ablation, *n* (%)	114 (64.0)	65 (78.3)	0.02
CTI ablation, *n* (%)	10 (5.6)	4 (4.8)	>0.9999
CFAE ablation, *n* (%)	17 (9.6)	10 (12.1)	0.52
Successful PWI, *n* (%)	9 (5.1)	5 (6.0)	0.77
Convert to AT, *n* (%)	15 (14.0)	7 (13.3)	>0.9999
Convert to sinus rhythm, *n* (%)	48 (27.0)	21 (25.3)	0.88
Cardioversion, *n* (%)	130 (73.0)	62(74.7)	0.88

Data presentation includes mean ± SD or absolute numbers along with percentages (n%). VOM refers to vein of Marshall; RF indicates radiofrequency; VOM-EI is an abbreviation for VOM ethanol infusion; PVI means pulmonary vein isolation; CPVA refers to circumferential pulmonary vein antrum; LA is short for left atrium; MI stands for mitral isthmus; CS indicates coronary sinus; CTI is cavotricuspid isthmus; CFAE denotes complex fractionated atrial electrogram; PWI refers to posterior wall isolation.

### 3.3 Clinical outcomes

**Figure 4:**
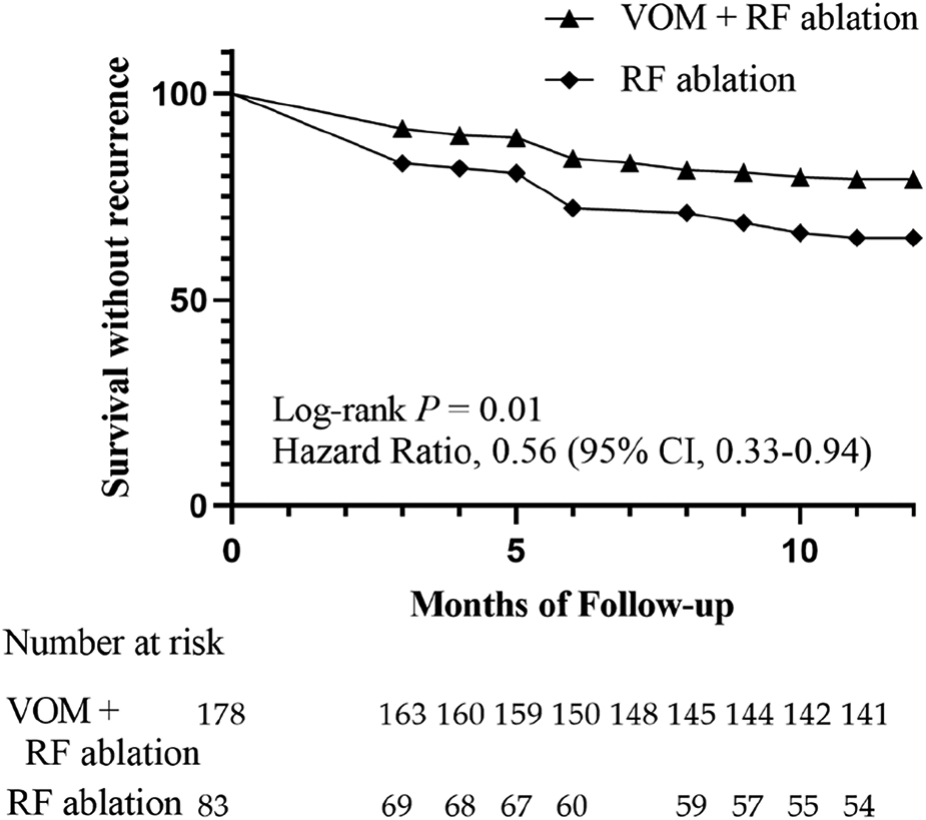
Twelve-month survival curve for patients without recurrence of AF.

According to Figure 4, the Kaplan–Meier curve indicated that the VOM + RF ablation group had a notably higher rate of recurrence-free survival compared to the RF ablation group [Hazard Ratio, 0.56 (95%CI, 0.33–0.94); Log-rank *P* = 0.01]. The VOM + RF ablation group achieved a survival rate of 78.7% (140/178) without recurrence, while the RF ablation group had a slightly lower rate of 65.1% (54/83). The VOM + RF ablation group had a lower recurrence rate with AT compared to the RF ablation group [8.4% [15/178] vs. 19.3% [16/83], *P* = 0.01], while the recurrence rate with AF was similar [16.9% [30/178] vs. 18.1% [15/83], *P* = 0.86]. The utilization of AAD post-procedure showed no notable disparity between the two groups, as 34.8% (62/178) in the VOM + RF ablation group was comparable to 37.3% (31/83) in the RF ablation group (Table 3).

**Table 3:** Clinical outcomes.

Variables	VOM + RF ablation (*n* = 178)	RF ablation (*n* = 83)	*p*-value
Survival without recurrence, *n* (%)	140 (78.7)	54 (65.1)	0.02
Recurrence, *n* (%)	38 (21.3)	29 (34.9)	0.02
Recurrence with AF, *n* (%)	30 (16.9)	15 (18.1)	0.86
Recurrence with AT, *n* (%)	15 (8.4)	16 (19.3)	0.01
Use of AAD post-procedure, *n* (%)	62 (34.8)	31 (37.3)	0.78

Data presentation includes mean ± SD or absolute numbers along with percentages (n%). VOM, vein of Marshall; RF, radiofrequency; AF, atrial fibrillation; AT, atrial tachycardia; AAD, antiarrhythmic drug.

### 3.4 Adverse event

A similar incidence of adverse events was observed between the VOM + RF ablation group and the RF ablation group, 2.25% (4/178) vs. 2.41% (2/83) (*P* > 0.9999). An arteriovenous fistula and pericardial tamponade were only observed in the VOM + RF ablation group, all in one patient. Only one stroke occurred in the RF ablation group (Table 4).

**Table 4:** Adverse event.

Complications	VOM + RF ablation (*n* = 178)	RF ablation (*n* = 83)	*p*-value
Pericardial tamponade, *n* (%)	1 (0.56)		>0.9999
Arteriovenous fistula, *n* (%)	1 (0.56)		>0.9999
Hematoma, *n* (%)	2 (1.12)	1 (1.20)	>0.9999
Stroke, *n* (%)		1 (1.20)	0.32
Total complications, *n* (%)	4 (2.25)	2 (2.41)	>0.9999

Data presentation includes mean ± SD or absolute numbers along with percentages (*n*%). VOM, vein of Marshall; RF, radiofrequency.

## 4 Discussion

### 4.1 Main findings

This study included a large cohort of patients with severe LA enlargement who underwent ablation to treat PsAF. This is a purely observational study, aiming to explore the correlation rather than establish a causal relationship. However, through our research, we still made some significant findings. The fundamental discoveries consist of (1) The VOM-EI assisted in the left CPVA and the MI ablation; (2) The VOM-EI enhanced the effectiveness of AF ablation; (3) AF ablation with VOM-EI notably decreased AT occurrence. These discoveries have, in fact, been reported in previous studies by others. However, our originality lies in the fact that we observed these findings in patients with severe LA enlargement without considering any factor other than LA diameter.

### 4.2 AF ablation in patients with severe LA enlargement

LA enlargement was associated with the morbidity and prognosis of AF.LA enlargement may increase the risk of AF recurrence following ablation, according to previous studies (4, 12). Furthermore, research has indicated that individuals diagnosed with PsAF exhibit an enlarged LA in comparison to those suffering from paroxysmal AF (13). These findings suggest that catheter ablation for PsAF in patients with LA enlargement has a poor prognosis. PVI and LA linear ablation are currently widely recommended traditional strategies for treating PsAF (1, 2). Recent clinical trials and retrospective studies have found that VOM-EI, combined with the conventional ablation strategy described above, can improve clinical outcomes (11, 24). However, whether VOM-EI improves the ablation outcome for PsAF in patients with severe LA enlargement is unclear.

An earlier study (25) found that the size of the LA was an independent predictor of AF recurrence after PVI. For every 1 mm increase in the LA diameter, the probability of AF recurrence increased by 7.2%. The anterior-posterior diameter has long been a commonly used indicator for evaluating the size of the LA in echocardiography. An LA anterior-posterior diameter greater than 45 mm is considered to have moderate or severe enlargement. In this study, we used a LA anterior-posterior diameter greater than 45 mm as the inclusion criterion for severe LA enlargement. This indeed was not sufficient to comprehensively evaluate all patients with LA enlargement. However, the results of this study have positive implications for assessing the prognosis of AF ablation in patients with a significantly enlarged LA. While LA diameter remains widely used in clinical practice, contemporary evidence suggests that LA volume and functional parameters may better reflect the severity of atrial myopathy. A recent study has found that patients with long-standing PsAF have experienced similar degrees of reduction in LA volume after undergoing catheter ablation or surgical ablation. The atrial contraction strain at 3 months is the most crucial determinant for AF recurrence after ablation (26). If LA volume and function assessment are incorporated in future studies, it will contribute to a deeper understanding of the specific impact of VOM-EI on PsAF patients with severe LA enlargement.

### 4.3 The facilitation effect of the VOM-EI in AF ablation

Studies have found that VOM is present in up to 95% of individuals. Anatomically, VOM connects the proximal end of the CS vein to the LA wall, concomitantly with the epicardium's Marshall ligament (MB). The main vessels can extend from the LA ridge to the roof of the left superior pulmonary vein and the LA wall. Local myocardial connections, nerves, and triggers can be ablated by retrograde infusion of the VOM, which can be used as a target for AF ablation (10). In our study, through voltage mapping, it was found that the VOM-EI caused a large LVA region in its distribution area, indicating that local ablation was achieved. The VOM-EI was linked to reduced ablation duration of the left CPVA and the MI, increased rate of MI block, and decreased CS vein ablation. This facilitation effect on the left CPVA and the MI ablation is consistent with the anatomical characteristics of the VOM. In a clinical trial and a retrospective study, it was found that the VOM-EI facilitated the ablation of the left CPVA and the MI (11, 24). In the VENUS trial (11), the success rate of MI block achieved by patients who received VOM-EI was 74.0%, and the duration of MI ablation was 8.1 ± 9.1 minutes. In contrast, the success rate of the MI block in our study was higher, reaching 94.9%. However, the duration of MI ablation for patients who received VOM-EI in this study was longer (23.2 ± 10.8 minutes), suggesting that more thorough ablation might be associated with a higher success rate of MI block. In this study, the success rate of MI block among patients who received VOM-EI was similar to the results of a retrospective study by Lai et al. (24), being 94.9% vs. 95.5%. Our study is the first to report that the effect of the VOM-EI, which facilitates AF ablation, has also been observed in patients with severe LA enlargement. Despite the fact that VOM-EI resulted in longer overall procedure and fluoroscopy durations, it notably alleviated the complexity of the left CPVA and the MI ablation. The findings indicate that the VOM-EI could potentially be beneficial in managing PsAF in individuals with severe LA enlargement.

### 4.4 Improvement of AF ablation outcomes by the VOM-EI

In the AF ablation field, PVI is considered to be the cornerstone. However, patients with PsAF are often associated with atrial fibrosis, and substrate modification beyond PVI may be necessary. A clinical trial compared the clinical outcomes of PVI with PVI plus substrate modification in patients with PsAF. At one year of follow-up, 71.3% of patients from the PVI group and 78.3% from the PVI plus group without AF recurrence (5). In our study, patients from the RF ablation group had a relatively low recurrence-free survival rate (65.1%) during the one-year follow-up, possibly due to the severe LA enlargement. However, adding the VOM-EI to the CPVA and linear ablation significantly improved the recurrence-free survival rate during the one-year follow-up, reaching 78.7%. In the VENUS trial, the recurrence-free survival rate in the VOM-EI plus catheter ablation group compared with the catheter ablation group was significantly higher (49.2% vs. 38%) (11). The ablation success rate reported in the VENUS trials is the success rate without AAD, which seems relatively low. In our study, 37.3% of patients from the RF ablation group and 34.8% from the VOM + RF ablation group were treated with AAD after the procedure, which may be associated with higher recurrence-free survival. The mechanisms by which the VOM-EI improves the success rate of AF ablation are likely complex, including the elimination of triggers (27), atrial denervation (28), and more reliable MI blocking (29). Left atrial tachycardias (ATs) are often seen after PVI with or without LA liner ablation. It has been found that 30.2% of the left ATs after AF ablation are MB-dependent, and VOM-EI should be implemented in these patients (30). The VOM + RF ablation group had a significantly lower recurrence of AT in our study compared to the RF ablation group, possibly due to the VOM-EI.

While the VOM has been increasingly recognized as a critical arrhythmogenic substrate in PsAF, recent imaging studies highlight that diverse anatomical variations may independently contribute to ablation failure. Recently, a study investigated atrial anatomical variations in patients with and without AF using cardiac computed tomography angiography (CCTA) and identified characteristics associated with AF recurrence after PVI. The study found that, compared with patients without AF, the prevalence of left atrial diverticulum, right atrial diverticulum, and Bachmann bundle shunt was higher in patients with AF (31). This research result indicates that pre-procedural CT/MRI may help identify patients who would benefit from extended substrate modification beyond VOM-EI.

### 4.5 Limitations

This investigation encountered several limitations. Initially, this was merely a retrospective observational study. Furthermore, defining LA enlargement based on diameter measured via echocardiography might overlook some cases with increased LA volume. Third, Holter ECG recordings during follow-up may need to be improved, and some recurrent cases may be missed. Fourth, the exclusion of patients whose VOM could not be identified or adequately treated may bias the findings. Therefore, our findings may only be applicable to patients with technically feasible VOM-EI; alternative ablation strategies should be considered for those who are not suitable for this approach. Furthermore, the follow-up lasted only a year, so extending it might provide more convincing results. Finally, this study only included patients from two medical centers, which might limit its applicability to a specific population. The results of this study still need to be verified more extensively, particularly in various healthcare settings.

## 5 Conclusion

The VOM-EI facilitated the left CPVA and the MI ablation and improved the ablation outcomes in patients with severe LA enlargement for treating PsAF.

## Data Availability

The raw data supporting the conclusions of this article will be made available by the authors, without undue reservation.
